# Short-Term Solutions to a Long-Term Challenge: Rethinking Disaster Recovery Planning to Reduce Vulnerabilities and Inequities

**DOI:** 10.3390/ijerph17020482

**Published:** 2020-01-11

**Authors:** Melissa L. Finucane, Joie Acosta, Amanda Wicker, Katie Whipkey

**Affiliations:** 1 Department of Behavioral & Policy Sciences, RAND Corporation, 4570 Fifth Ave, Pittsburgh, PA 15213, USA; 2 Department of Behavioral & Policy Sciences, RAND Corporation, 1200 South Hayes St, Arlington, VA 22202, USA; jacosta@rand.org; 3 Department of Engineering and Applied Sciences, RAND Corporation, 1200 South Hayes St, Arlington, VA 22202, USA; awicker@rand.org; 4 CARE Nederland, Parkstraat 19, 2514 JD Den Haag, The Netherlands; katiewhipkey@gmail.com

**Keywords:** disaster recovery, post-disaster policies, social vulnerability, inequity, decision making

## Abstract

In the immediate aftermath of disaster, governments usually act quickly to reduce risk and to recover their communities’ socio-economic functioning. Policy makers in these situations need—but may not have the capacity or time for—substantial analysis and public debate about how to balance short- and long-term societal needs. Inadequate attention to this challenge may result in a deepening of the inequities that increase vulnerability to disaster impacts. We review case examples to illustrate how post-disaster policies may influence the nature, pace, and inclusiveness of community recovery. We then apply a vulnerability/inequity framework to conceptualize how to enhance disaster recovery and avoid perpetuating inequities when weighing the diverse needs of communities across long time horizons.

## 1. Introduction

Disasters are becoming more common and costlier [1,2]. Flooding and storms are the most frequent types of disaster and earthquakes (including tsunamis) are the most deadly [3]. During 1994–2013, the Emergency Events Database (EM-DAT) recorded 6,783 natural disasters worldwide, claiming on average almost 68,000 lives per year and affecting 218 million people each year. Higher-income countries experienced 56% of natural disasters but lost 32% of lives; lower-income countries experienced 44% of disasters but suffered 68% of deaths [2]. Globally, 315 natural disaster events were recorded in 2018, causing 11,804 deaths, affecting over 68 million people, and resulting in US$131.7 billion in economic losses; Asia suffered the highest impact [3]. From 2000 to 2018, the U.S. experienced 58 major declarations per year on average and a total of 2,351 declarations overall [4].

Environmental and demographic trends are leading to more extreme disasters and associated socio-economic trauma, especially for more vulnerable populations (e.g., lower socio-economic groups, children, older adults) [5]. Coastal inundation is expected to worsen in many places before the end of the century because of rising sea levels due to the changing climate [6,7]. However, global population growth and patterns of economic development are relatively more important than climate variation or change in explaining the upward trend in climate-related disasters [2]. In addition, many communities face chronic economic stress. For instance, the richest 10 percent of the US population make over nine times more than the poorest 10 percent, resulting in the highest level of income inequality in the past 50 years [8]. Compared with advanced economies, however, income inequality is typically higher in developing and emerging economies [9].

Policy makers must address the changing nature of risk exposures and social-ecological sensitivities, without deepening inequities. However, the nonlinear and multidimensional nature of disaster recovery means that urgent pressures can give rise to reactive policies that fail to address (or might even amplify) key drivers of vulnerability. The purpose of this article is to review case examples to illustrate how post-disaster policies may influence vulnerability, inequity, and the nature, pace, and inclusiveness of community recovery. We then discuss ways in which disaster recovery planning activities, including elements of disaster governance, might reduce vulnerabilities and inequities.

**Figure 1:**
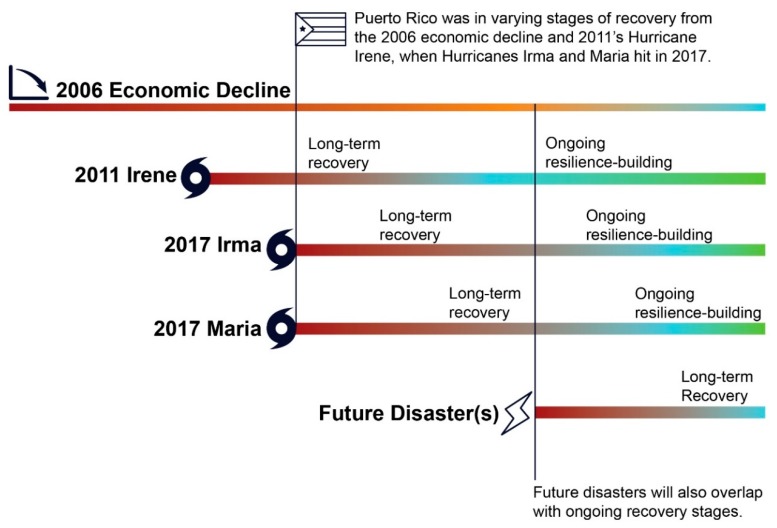
The recovery continuum: overlapping disasters complicate recovery processes (Adapted from [11])**.**

When multiple disasters occur in quick succession, periods of response, recovery, and preparation for future risks may overlap or take longer than originally expected. For example, Puerto Rico was still recovering from Hurricane Irene in 2011 and a long period of economic decline when Hurricanes Irma and Maria hit in 2017 (see Figure 1). Planning for recovery in the context of overlapping or compounding disasters [10] requires substantial analysis and public debate of difficult tradeoffs as decisions are made about infrastructure repair, economic development, environmental cleanup, restoration of natural systems, urban redevelopment, hazard mitigation, equity and justice, and other challenging issues.

A central challenge inherent in disaster recovery is addressing short-term needs quickly, yet with enough foresight to avoid creating new or worsening existing long-term societal needs that can contribute to a community’s vulnerability to future disaster. On one hand, a speedy approach is important for keeping businesses operating, providing temporary and permanent shelter for disaster victims, and rebuilding infrastructure that is important to community functionality and the economy. On the other hand, systematic and inclusive deliberation across governmental levels and with the public is an important part of post-disaster reconstruction planning. Inclusive deliberation helps to ensure that land use and infrastructure are coordinated and safe, that approaches to rebuilding improve residents’ quality of life, that the needs and concerns of all citizens are heard, and that cost-effective solutions to recovery efforts are identified. For successful long-term recovery, inclusive deliberation and planning requires relevant and up-to-date scientific data as well as sharing of information across agencies and sectors, so that alternative paths forward can be evaluated, and robust solutions can be developed. Unfortunately, the urgent, short-term pressures in the aftermath of disasters sometimes mean that longer-term vulnerability reduction is overlooked as a fundamental component of recovery.

### Theoretical Framework

Emerging work on disaster resilience, risk reduction, and governance has provided multiple overarching frameworks for the discussion of issues related to disaster recovery, vulnerability, and inequity [12]. Principles of disaster risk reduction and management [13,14,15,16] emphasize that post-disaster recovery and development need to be informed by effective resilience building and community engagement and empowerment strategies. A unique and defining characteristic of the recovery period is “time compression” [17]. That is, during recovery, community development activities that traditionally take place over time are instead compressed to a limited space (i.e., places damaged by disaster) and time period. This time compression and the role of various organizational, institutional, and other social forces, must be considered in approaches to disaster preparedness and recovery planning activities [18,19,20]. Otherwise, the focused space of a disaster will deepen differences in access to scarce resources and power and disproportionately affect capacity to cope with long-term impacts. Early decisions may prevent longer-term vulnerability reductions once recovery efforts are underway.

**Figure 2:**
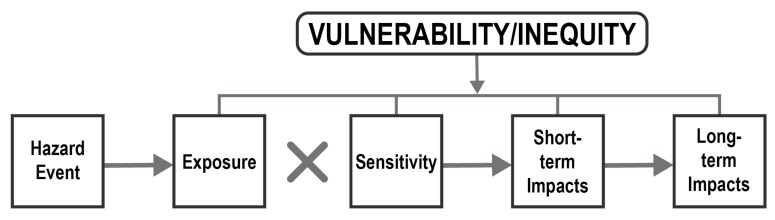
Simplified vulnerability and inequity conceptual framework (adapted from [41]).

The term “vulnerability” is used in a variety of ways in the disaster risk literature [21,22]. In studies of disasters and disaster risk reduction, vulnerability is commonly defined as “a measure of the propensity of an object, area, individual, group, community, country, or other entity to incur the consequences of a hazard” ([23], p. 33). The term “inequity” is commonly defined as “that which disproportionally favors well-off groups over those in unfavorable and vulnerable conditions” [24]. Inequities in the distribution of resources (e.g., federal disaster aid) or social opportunities can lead to recovery outcomes that disproportionally favor those already in power or who have access to economic and other resources, compared with those in socially vulnerable populations (e.g., minorities, female-headed households, low-income households, the elderly) [25,26,27,28]. Vulnerability and inequity are intrinsically related to each other and to the social dimensions of disaster, disaster risk, and disaster impacts [12,29,30,31,32,33,34,35,36]. When combined with assessments of hazards, assessments of vulnerability and inequity can inform a community about their overall risk [37,38]. Figure 2 shows a simplified conceptual framework, which posits that vulnerability and inequity influence an individual’s or system’s exposure to specific hazards, increases their sensitivity to hazards, and therefore some individuals and systems are more likely than others to be impacted by (and less resilient to) specific hazards. Following prior work (e.g., [36,39,40,41]) exposure refers to who or what is at risk, sensitivity is the degree to which harm may occur to people and places, and impacts are the direct and indirect consequences of a disaster event.

Community resilience frameworks like the Hyogo Framework for Action [15], a global framework to reduce disaster losses and improve lives after disaster, have characterized vulnerability as a pre-disaster issue to be assessed and mitigated [15]. Similarly, the National Disaster Recovery Framework views recovery planning as an opportunity to reduce vulnerability to hazards [42]. Recent approaches to emergency management recognize that any person can experience vulnerability, especially following a disaster. For example the C-MIST framework used by the Federal Emergency Management Agency (FEMA) and the Assistant Secretary for Preparedness and Response identifies the following types of circumstances that can make people more vulnerable in a disaster: Communication challenges, health Maintenance needs or reliance on medical devices or supplies to remain Independent, Services to manage behavioral health needs or infants and children who rely on others for sustenance, and Transportation obstacles [43]. These approaches are limited in that they focus primarily on how pre-disaster conditions influence impacts post-disaster, and do not consider how post-disaster decision making and other social forces can impact vulnerability and inequity over time.

In contrast, the Sendai Framework for Disaster Risk Reduction [16], which builds on the Hyogo Framework for Action, broadens the concept of disaster risk reduction to include post-disaster recovery and development activities to encourage recovery plans to focus on restoring and rebuilding in ways that mitigate or reduce future vulnerabilities [16]. The Sendai Framework has an explicit goal to “build back better” and references the importance of broad-based collaboration to achieve this goal. One of this framework’s priorities for action—disaster governance—is a coordination mechanism for collaboration and spans the phases of disaster management from preparedness to long-term recovery [12,32,34,44,45]. Tierney [12] defines disaster governance as “the interrelated sets of norms, organizational and institutional actors, and practices… designed to reduce the impacts and losses associated with disasters” (p. 344). In contrast to the typical top-down, command-and-control emergency-centric approach, disaster governance emphasizes: engaging multiple actors at multiple levels; building trust and social capital through collaboration and leadership; learning and innovation; and building strong formal and informal networks through bridging and boundary spanning organizations [46]. A core feature of disaster governance is social inclusion, that is, engaging a broader constituency of local society in decision processes about disaster response and recovery in the short- and long-term [45]. These adaptive governance elements are a crucial way to achieve the multidisciplinary and transdisciplinary approaches in research and practice that are necessary for more equitable, resilient, and sustainable development. However, disaster management has been challenged by global disparities in income, well-being, and political empowerment as well as growing disaster vulnerabilities across the globe. The present paper explores case examples from past disasters that illustrate some of the challenges to and implications for planning and governance to better mitigate vulnerabilities and inequities during recovery.

## 2. Materials and Methods

### 2.1. Identify Case Examples from Past Disasters

We first identified case examples of how policies might aim to reduce vulnerability by decreasing exposure in the short term, but in doing so may exacerbate factors contributing to sensitivity, which can increase vulnerabilities and inequities in the long term. To identify examples, we searched peer-reviewed literature, policy documents, and recovery plans from three recent disaster recovery efforts in island communities: New Zealand, Sri Lanka, and Puerto Rico. Although these contexts differ in many ways (e.g., along geographical, socio-cultural, governance, and economic dimensions), they are similar in the close proximity of their residents and infrastructure to coastal areas. Recovery is challenging in a context where lifeline sectors or systems (energy, water, transport, telecommunications) are spatially concentrated and could produce cascading failures and have a debilitating effect on public health, safety, and economic security [47,48]. Extreme water-level events (caused by hurricanes, tsunamis, or earthquakes) pose a serious risk of inundation to island communities, which in turn can damage freshwater sources and supplies, agriculture, physical infrastructure, and important ecosystem services provided by coastal areas. The geographical isolation of these island settings also complicates disaster response and recovery efforts, in part because of the time and cost of transferring aid from other jurisdictions. Given the challenges, these island communities offer an excellent opportunity to examine how short-term recovery policies may unintentionally increase longer-term vulnerability. Nonetheless, focusing only on these cases does limit the generalizability of findings to other contexts. In the examples below, we highlight one specific policy or decision implemented in each community as a result of a major disaster and discuss its impact on vulnerabilities and inequities. These policies were intentionally selected because they had implications for vulnerable populations: a short-term decrease in exposure may have occurred, yet the long-term impacts on vulnerability and inequity were negative. Although long-term impacts in the Puerto Rico case example are still unknown, we included this case because of its timeliness and because prior research suggests that a negative impact on vulnerability and inequity is likely.

### 2.2. Organize Case Examples by Interacting Components of Vulnerability and Inequity

To understand why some people are more vulnerable than others (and why some communities recover more quickly than others) from disasters, we need to consider the historical background and socio-economic conditions in the communities, and how the long-term consequences of disasters compound as a result of this context. The recovery burden for some is greater than for others, but we cannot understand for whom or why without a broad analysis of the multiple factors affected by post-disaster policies. To illustrate the potential impacts of recovery policies, we organized the case studies according to relevant elements of vulnerability and inequity (but do not analyze the effectiveness of the policies, since that is beyond the scope of this paper).

## 3. Results

### 3.1. Canterbury Earthquake, 2011: Establishing the Canterbury Earthquake Recovery Authority, New Zealand

#### 3.1.1. Brief Policy Description

New Zealand’s approach to emergency management was guided by the Civil Defense Emergency Management (CDEM) Act 2002 and Canterbury Earthquake (Resource Management Act Permitted Activities) Order 2011, which outlined the structural and operational procedures of federal disaster response [49,50]. (The acts guiding emergency management are operational in nature; they do not provide details on elements of vulnerability or vulnerable populations.) The Canterbury Earthquake Recovery Authority (CERA; Te Mana Haumanu ki Waitaha) was established as a new governance structure formed about one month after the 2011 earthquake near Christchurch, New Zealand [43]. The policy centralized recovery authority and operations at the national level because prior to the establishment of CERA, no government entity had the power or time to manage national recovery efforts [51]. CERA was given wide-ranging powers (e.g., suspending laws and regulations, acquiring land), for a limited time, for the purpose of earthquake recovery. CERA was responsible for coordinating the recovery response in the greater Christchurch area, which includes the districts of Christchurch City Council, Selwyn District Council, Waimakariri District Council, and the nearby coastal marine area. Approximately seven percent of New Zealand’s Māori population lived in this region, accounting for over 40,000 people [52]. CERA’s responsibility involved: “providing leadership and co-ordination for the recovery effort; enabling an effective and timely recovery; monitoring the progress of the recovery; and administering the Act” ([53], p.13). In addition, CERA administered projects and programs including repairing and rebuilding infrastructure, deciding the future for land use, and supporting psychosocial recovery. The purpose of CERA was to facilitate complex rebuilding in a compressed time period through enhanced coordination among national agencies and expedited policy and decision making. As a result of CERA’s broad mandate, the authority became a “catch-all” agency, with its specific role becoming increasingly less clear as recovery progressed [53].

#### 3.1.2. Example Impacts on Components of Vulnerability and Inequity

Analysis by Johnson and Mamula-Seadon [54] suggests that creating CERA reduced exposure nationally by enabling a swifter response to disasters at a national scale, increasing communication across government levels, and facilitating long-term focus on recovery. At the same time, CERA both attenuated and exacerbated sensitivity in local communities. Where communities were already highly organized and mobilized, localized response enabled them to receive swifter and more comprehensive support than if they relied on a national response (i.e., in this instance, CERA slowed recovery and resulted in a narrower range of services). In other communities that lacked organized local support, CERA attenuated sensitivity [55,56]. For instance, the government offered to purchase properties from homeowners in the most damaged residential areas [57].

One criticism of CERA relates to its lack of vertical coordination, particularly regarding local collaboration and engagement [54]. Local and regional authorities raised concerns about decreased coping and long-term adaptation. They were concerned that a lack of involvement of important local partners in planning, implementation, and decision making may have led to delays [51] or perceived inequities in housing valuations (e.g., the residential red zone buyout program) [58]. For example, the residential red zone system developed red and green zones; red meaning lands susceptible to greater risk and green those with lower risk. Repairing red zone properties was determined to be too costly and risky, so the government and insurers bought-out those properties from owners and declared those areas uninhabitable [59]. One key issue with the red zone buyout program was that it only focused on those with enough wealth to own a home and left out over a third of the population. Those benefiting from the buy-out program were still often stuck between insurers and the government with little opportunity to contribute to the decisions affecting their lives, and CERA’s policies and procedures were not designed in a way to support this dilemma [60].

Social sensitivities were not fully understood by the CERA planning processes, increasing the vulnerability of cultural risk to Māori communities in particular. The land zoning decision making, for example, threatened Māori culture and language. The decision making was done scientifically based on risk measurements of hazard, vulnerability, exposure, and economic implications. Sense of place and cultural significance were not taken into account at the outset, though for some alternative components were incorporated eventually [61]. News reports often cited perceived inequities in CERA’s planning and consultation. Some sources felt that despite CERA officials often acting with good intentions, the “need for speed” made wider community consultation difficult and instead reinforced existing biases by executing consultations with those already “more or less exactly like themselves” [61]. Others were even more critical, feeling that the government was executing a “command and control” approach [62].

#### 3.1.3. Alternative Ways to Reduce Vulnerability and Inequity

When CERA was disestablished (as planned) in 2016, evaluations of its success were conducted by both the New Zealand Department of the Prime Minister and Cabinet and the Controller and Auditor-General. The Department of Prime Minister and Cabinet put forth several recommendations, in case another disaster warranted a similar authority. One recommendation was to engage the community in every step of the process from planning to implementation to review, and to clearly communicate how the community inputs informed the plan. For example, as New Zealand’s tangata whenua (people of the land), Māori stakeholders have key cultural, spiritual, historic, and environmental perspectives. These perspectives, inclusive of local and/or indigenous knowledge, need to be incorporated better into long-term planning and decision-making processes as they expand the pool of potential solutions [63]. Reducing vulnerability requires a fluid, heterogeneous, and dynamic process [61]. Adaptive governance may be a mechanism to improve collaborative planning and reduce vulnerability and inequity [46]. Scholars have proposed four characteristics of adaptive governance: polycentric and multilayered institutions alongside leadership, trust, and social capital; community participation and collaboration; self-organization (formal or informal) and networks; and learning and innovation [64]. Thus, should an entity like CERA need to re-emerge, these aspects should be built into the agency’s operating procedures from the start and evaluated throughout its operating cycle.

Another recommendation from the evaluation was to create agreements between local agencies to determine how services can be collaborative and streamlined to minimize confusion and delays post-emergency. Vulnerability could be reduced further by building in more safeguards to ensure the authority’s power cannot be abused, including putting legislation through more rigorous processes rather than enabling an authority to fast-track decision making in ways that may increase long-term vulnerability and inequity. A future authority like CERA should clearly document the responsibilities of all agencies to ensure accountability and to mediate the public perception of what the authority should and should not be completing [51,57]. This recommendation was echoed in the evaluation by the Controller and Auditor-General, who also emphasized the need for separating strategic from operational decision-making and reviewing the governance structure of responsibilities [53]. Finally, building in systems and processes that enable citizens to enhance their own resilience through information was recommended. Citizens stated that access to information (e.g., Geonet, QuakeLive) enhanced their personal decision-making ability [61]. A more holistic policy that incorporates best practices of disaster planning and recovery is warranted to decrease vulnerability and inequity.

### 3.2. Indian Ocean Tsunami, 2004: Coastal Buffer Zone Policy, Sri Lanka

#### 3.2.1. Brief Policy Description

Within days of the 2004 Indian Ocean tsunami, the government of Sri Lanka implemented a coastal buffer zone policy to prevent people from moving back to risk-prone areas [65]. The policy prohibited repair or reconstruction of homes (regardless of the percentage of structural damage) within 100 m of the coastline in the south and west and 200 m in the east. The rationale for the extent of the zone was not entirely clear, but seems to reflect the level of damage observed in different areas, topography, and structure use (permanent or temporary) [66]. Initial estimates suggested that more than 60% of the houses damaged were within the buffer zone and thus required their inhabitants to relocate, despite their heavy dependence on coastal resources such as fishing for their livelihoods [65]. Intense opposition from local communities and businesses led to the government rescinding the initial buffer zone, relying instead on existing regulations from the 1981 Coastal Conservation Act. These regulations had not been enforced since the act was created but redefined the zone to being within 10–125 m of the coastline (a distance based on coastal erosion rather than tsunami impacts), depending on area topography and structure use (temporary or permanent).

In contrast, hotels with less than 40 percent structural damage were allowed to remain within the buffer zone, presumably because this was not considered permanent housing. Consequently, a process of gentrification was initiated in which hoteliers and the tourism industry expanded rapidly while fishing communities were marginalized [67]. In addition, reconstruction at government-designated resettlement sites outside the buffer zone was unevenly distributed and paced. Little attention to pre-existing development regulations, land-use policies, or the social impacts of resettlement [68] resulted, in some instances, in inequities in the rebuilding of homes and livelihoods [69,70]. For instance, while rapid construction of resettlement camps two kilometers from the coast was considered a success by some, fisher-folk relocating to such sites were very concerned about how they would continue fishing and did not see options for alternative livelihoods [71]. Another example is that the post-tsunami reconstruction boom created temporary prosperity for some (e.g., semi-skilled laborers) but rendered goods and services unaffordable for others [70]. Ultimately, land scarcity issues resulted in the government revising this policy decision. However, Ingram [71] et al. [71] found that even a year after the tsunami, many affected communities were still unclear about where building was allowed or where it would proceed.

Since the tsunami, the Sri Lanka Disaster Management Act No. 13 of 2005 established the National Council for Disaster Management to provide high-level oversight for the different line ministries involved in all phases of the disaster timeline. The main implementing body for the Council is the Disaster Management Center. Review of the country’s main governing policy for disaster risk reduction—the Sri Lanka National Disaster Management Policy (SLNDMP)—suggests that improvements are needed to more closely align it with principles of the Sendai Framework. For instance, the SLNDMP does not require post disaster assessments, which would be invaluable for informing mitigation activities [72].

#### 3.2.2. Example Impacts on Components of Vulnerability and Inequity

Analysis of impacts of the coastal buffer zone policy [71] suggest that although exposure to coastal inundation was swiftly reduced, long-term sensitivity was increased in some groups. First, pre-tsunami socio-economic disparities were amplified (e.g., people of lower socio-economic means became more reliant on the informal economy and less likely to have land titles, insurance, or bank accounts) [73]. In some locations, cash-for-work programs (e.g., clearing rubble and building transitional shelters) enabled affected people to obtain employment. However, such programs largely benefit able-bodied people, particularly men. Older adults, sick or disabled, and carers (typically females) are less able to benefit [65]. In another example, an estimated 15,000 survivors were involved in informal sector activities (e.g., food processing, coir manufacture, tailoring) pre-tsunami, but relocation to places further from necessary resources, equipment, and/or markets make dependence on such activities less reliable. Since these types of livelihoods are often more important for women, female headed households (about a sixth of affected households) may recover more slowly.

Cost increases in the construction industry—a result of increased demand for construction inputs—make the task of reconstruction much more difficult for poorer households. While more affluent households with access to savings and relatively cheap credit can draw down their savings or cut back on consumption to finance the necessary additional expenditures, poorer households lack savings, access to cheap credit, and capacity to repay. When poorer households struggle to make progress with construction tasks, they face increasing difficulty in maintaining their eligibility for subsequent instalments of aid. Without substantial additional assistance, rehabilitating damaged houses and other assets is extremely hard for poorer households. In addition, people living in transitional shelters report poorer health compared with people relocated to housing projects, adding to their burden and increasing relative vulnerability [74].

For people who formerly relied on coastal resources such as fisheries, livelihood opportunities at the more inland relocation sites were limited. In urban and densely populated areas, relocation of business-related buildings could be costly. Moreover, some fishing activities, such as the drawing in of large nets, require community participation. Community networks, however, were severely disrupted by non-traditional groupings of socio-economic and cultural classes in resettlement camps. These changes impacted not only the relocated population, but also negatively impacted the overall economy of Sri Lanka [73].

Overall, Sri Lanka’s coastal buffer zone policy demonstrates a reactive adjustment that did not enhance long-term adaptation, but rather resulted in an inequitable rebuilding of homes and livelihoods and reduced the resilience of some communities through reduced coping [71]. Post-tsunami livelihoods are inextricably intertwined with shifting labor needs, market priorities and demands, and the influx of international humanitarian aid organizations in affected areas [70]. Unfortunately, more vulnerable groups that depend on fisheries and tourism-related informal sector activities bear the brunt of the negative impacts [73].

#### 3.2.3. Alternative Ways to Reduce Vulnerability and Inequity

While short-term recovery efforts must urgently address the need for safe shelter and livelihood security, long-term redevelopment policies should be developed more cautiously, with input from comprehensive, place-based assessments and consultation with local stakeholders [71]. Adaptive governance may be helpful in this context to improve collaborative planning [46]. Recommendations to decrease vulnerability might include protection and management of environmental resources (e.g., sand dunes, coral reefs, mangroves) to reduce physical exposure to waves and storm surges, but also to decrease sensitivity by protecting a vital source of food and income for local people. In addition, sensitivity could be decreased by enhancing opportunities for lower socio-economic groups to access financial resources (e.g., micro-finance institutions and insurance measures), providing secure access to land and natural resources to stabilize livelihoods, and increasing technical and logistical assistance for finding livelihood alternatives.

The extent to which these strategies are practical or achievable in Sri Lanka within existing institutional structures is uncertain. For instance, a lack of government capacity to deal with compensation claims within the fisheries sector resulted in substantial delay. In an attempt to fill this gap, humanitarian aid organizations mobilized large, but uncoordinated amounts of funding, providing ad hoc assets (e.g., motorized boats) that could result in more degradation of available fish stocks and ultimately undermine livelihoods in the long term and fuel community tensions [65,70]. Long-term recovery requires multi-year and cross-sectoral planning and policies to support sustainable management of coastal resources. Such efforts are underway in Sri Lanka, but are still a relatively new endeavor for a country with no previous history of a similar disaster.

### 3.3. Hurricane Maria, 2017: Permanent Closure of Public Schools, Puerto Rico

#### 3.3.1. Brief Policy Description

**Figure 3:**
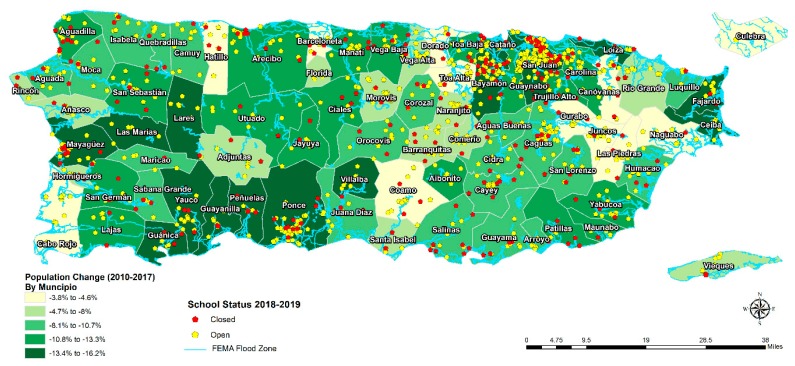
Puerto Rico’s school closures and change in population (2010-2017) (courtesy of the Center for Puerto Rican Studies at Hunter College, City University of New York [81]).

Prior to the 2017 hurricane season, about 167 of roughly 1300 public schools in Puerto Rico were closed because of the declining number of school-aged children—approximately 20,000 students per year [75,76]. These closures were a continuation of a 2014 Puerto Rico Department of Education policy, “Plan for School Transformation and Reorganization,” that aimed to combat declining registration, ineffective investments, and poor academic performance [77,78,79]. The damage and destruction caused by Hurricanes Maria and Irma only exacerbated the ongoing issues with school infrastructure, declining registration, and debt. In the months after the hurricanes made landfall on the Island, Governor Roselló announced a new fiscal plan [76] which, based on an expected decline of 27,500 students and a drop of 7300 teaching staff [80], expanded the consolidation efforts and ultimately resulted in the permanent closure of more than 250 schools (totaling approximately a quarter of the schools at the time) (see Figure 3). The government of Puerto Rico proposed that the school closures would increase the quality of education provided to students and allow more funds to be directed to the remaining schools [75].

#### 3.3.2. Example Impacts on Components of Vulnerability and Inequity

Closing schools swiftly after Hurricane Maria reduced exposure for students and teachers, for instance to structural damage, mold, and vermin. However, leaving these school buildings abandoned, rather than repurposing them, may have increased the opportunity for them to shelter illegal activities such as drug-related violence and other crimes [82]. Moreover, longer-term increases in vulnerability may result from the policy increasing sensitivity through several factors. For instance, closing schools could prompt even more students to relocate, compounding the economic problems in Puerto Rico. Very little research has been done so far to examine the impacts of school closings in Puerto Rico, which is especially difficult to determine given the lag in economic indicators. However, research in other US locations suggests that consolidation would produce fewer overall financial benefits than expected (e.g., because student transportation costs increase) and negatively impact the local economy in poor and minority communities where schools are often closed [83].

A second example of how the school closings can exacerbate inequities is by disproportionately impacting the economies and families in vulnerable communities. Sixty-five percent of school closures occurred in rural areas compared to urban areas [81]. This results in an increased burden of travel to school falls disproportionately on rural, poor, and isolated families [84] when their new schools are further away and roads are less accessible [85,86]. Expecting remote communities to relocate to larger towns poses additional challenges such as financial burden, employment instability, psychological distress, and loss of social capital. Additionally, teacher attrition and increased student classroom sizes, which may come as a result of the consolidation, could lead to a decrease in the quality of education and have a negative impact on the performance of students from closed schools [82,87]. This is critically important as student performance can have a profound impact on employment, earning, and quality of life as an adult [88].

A third way in which inequities may increase is by a disproportionate impact of the closures on younger students or others requiring assistance for disabilities [89]. In order to determine which schools would be closed, the Puerto Rico Department of Education conducted an analysis they stated was based on factors including enrollment and capacity, facility quality, distance and transportation, school performance, services for special education students, and community input [75]. However, the Puerto Rico Civil Rights Commission concluded that the Department’s school closures’ process neither consistently weighted their stated criteria in the analysis, nor appropriately considered other key factors. In particular, the Commission noted that the analysis failed to consider the impact on students who participate in special education or are socioeconomically disadvantaged. Although the Department of Education claimed that they considered suggestions from students, parents, and the community at large, they ultimately rejected their input, effectively excluding them from the school closures decision process [84].

Finally, permanently closing schools can increase long-term vulnerability by eliminating important community hubs and removing trusted adults (teachers) from their daily interaction with students, thereby reducing opportunities for community coping [90] needed to overcome trauma [91]. Research shows that social networks are often built around schools and their absence decreases the overall sense of community and local response capacity [92]. Erosion of key social networks can also result in a lack of meaningful engagement in decision making within local communities, decreasing coping and long-term adaptation at the local level [84,93,94,95].

#### 3.3.3. Alternative Ways to Reduce Vulnerability and Inequity

During the transition between disaster response and recovery, it is critical to provide communities with consistent support and to consult with and continually inform them as longer-term plans are developed. Such practices are valuable in any context, but particularly important following a disaster. The Puerto Rico Civil Rights Commission recommended that the Department of Education carry out a phased transition that allows for consultation with and participation of a broader array of community stakeholders (e.g., students, families, educators, administrators, mayors) and an awareness campaign about the process for school closures and transition to receiving schools [84]. Adaptive governance could support these collaborations [46]. The Commission also recommended that the Department of Education develop a protocol for school closures that includes: the process of identification; the participation of students, the school community, and the community-at-large; the establishment of criteria; and a phased orientation of all affected parties. Furthermore, the economic and disaster recovery plan for Puerto Rico [96] recommends a longitudinal data system to help the Department of Education to make timely, evidence-driven decision making related to decentralization, resource allocations, and future school closures. Some impacts of the closures of elementary schools may be mitigated by the increase in public Montessori schools, particularly for those in rural communities [81].

## 4. Discussion

The case examples described above illustrate that diverse actors in communities need support as they face complex disaster recovery decisions that have both short- and long-term implications for health and equity. The examples also suggest what types of support are likely most important when the goal is to reduce vulnerability and inequity. Responding in the short term and long-term efficiently and cautiously, requires the mobilization of tools that directly reduce social-ecological sensitivities and facilitate informed and effective recovery efforts. Below we describe preliminary ideas about two sets of tools that address these objectives. Importantly, discussion of these proposed tools is nascent; whether the tools would be sufficient or whether changes in disaster governance would be needed to ensure effective adoption of the tools will require systematic and iterative evaluation.

### 4.1. Adaptive Disaster Recovery Planning

The first set of tools is best described as adaptive disaster recovery planning. The example tools discussed below are the support function-based system and adaptive post-disaster financing. These tools are necessary to ensure that diverse community needs are understood and addressed and to permit course corrections as circumstances change.

Since the range of disaster impacts is typically broad, communities must come together collectively to recover; collaboration among a diverse array of governmental and nongovernmental actors is needed [16,97]. Such collaboration is consistent with the gains in efficiency expected by a whole community approach, as highlighted in the Sendai Framework. The coordinating structure for key functional areas of assistance in the National Disaster Recovery Framework are “recovery support functions” for each main sector (economic; health and social services; housing; infrastructure; natural and cultural resources; community planning and capacity building). The recovery support functions aim to support local governments by facilitating problem solving, enhancing access to resources, and fostering coordination among actors. Shifting from an incident command structure often used in response to an emergency, to a support function-based system in recovery has been shown to provide more opportunity for inter-organizational relationships, but may be derailed if there has been insufficient time to build trust and common communication processes [98]. Overall, multiple stakeholder perspectives need to be integrated to support disaster recovery [99]. Given the emphasis that disaster governance frameworks place on collaboration, more research is needed on tools that best support the positive drivers of disaster governance. Positive drivers in the pre-disaster period include legal and financial incentives, strong political leadership, local governmental capacity to support implementation of risk mitigation activities, information sharing to raise public awareness, and public participation processes to strengthen accountability [100,101].

In addition to enhancing responsiveness, recovery planners need to be adaptive as circumstances evolve. New tools for providing relief (e.g., adaptive post-disaster financing) [102] might allow more flexibility to address changing conditions as recovery processes progress. Adaptive post-disaster financing has been proposed to address the common community challenge of receiving too little recovery funding, at a time when the greatest needs have passed [103,104]. For instance, companies could have a set of pre-approved solutions based on different likely scenarios for their market (e.g., a 6-months interest free loan for storm damage) that could be adapted quickly after a disaster to meet needs of individuals and businesses. This tool adjusts to meet the complex community needs post-disaster by, for example, integrating complementary financial products (e.g., short-term loans) with non-financial programs (e.g., household-reconstruction planning) [102]. This tool would require private sector engagement and new funding models to be in place prior to a disaster, but could mitigate the impact on larger markets often created by supply chain disruptions post-disaster [102,105].

### 4.2. Formal Mechanisms for Assessing and Addressing Inequities

The second set of tools for reducing long-term vulnerabilities relates to formalizing mechanisms for assessing and addressing inequities in decision processes. Such tools may include equity impact assessment, social impact assessment, intensive technical assistance, and ethical debt instruments (e.g., green and blue bonds).

For instance, equity impact assessments could be required as part of permitting processes during rebuilding. Urban planners have used these assessments when planning for issues related to climate change, regional land-use planning, transportation, and road construction [85,106,107]. The assessments can be done at a micro-level (examining the impacts of specific policies or programs) or at a macro-level (assessing the mix of policies and programs) [108,109]. Assessing equity impacts aids the development of recommendations on how to mitigate any negative impacts and maximize positive impacts and how to improve programs or services to be more responsive to specific populations. Case studies have shown that equity impact assessments can change decisions, enhance plan content, and improve plan implementation [106,110]. However, the assessment must be conducted in a timely manner prior to a plan’s completion and have a clear definition of the scope, purpose, underpinning values, and what is hoped to be learned [111]. A shared understanding and agreement about the purpose of the assessment is needed at an early stage to ensure that decision makers and planners engage in a meaningful learning of equity issues that leads to the integration of changes to planning activities, rather than generating a separate plan [110,112].

Typical steps in an equity impact assessment include (1) screening to determine the suitability of a policy or program for inclusion in the assessment (e.g., what populations and what dimensions of equity are affected); (2) scoping out the dimensions of equity and identifying outcome measures that could be used for policy/program monitoring; (3) identifying impacts through various data collection techniques (e.g., literature review, consultation with stakeholders); (4) assessing impacts by weighting and analyzing the data collected and then reviewing the potential impacts with experts and stakeholders; (5) recommending changes to the policy or program based on likely equity impacts; and (6) continuing to monitor and evaluate of the uptake of recommended changes and the outcomes of the proposed policy or program [108]. These steps could be adapted and applied to key decisions in the recovery process. Some rapid equity impact assessments have been done in as quickly as four days to ensure timely information from the assessment is available for recovery decision making [111,113].

Social impact assessment (SIA) and the SIA Framework for Action provide a second type of tool that could be applied to a post-disaster context [33,114]. SIA is the process of managing the social issues associated with planned interventions (projects, programs, policies, plans) that may impact local communities [115,116]. Designed as an intentionally flexible process, SIA has four main phases that are somewhat sequential, but may also overlap: understanding the issues; predicting, analysing, and assessing the likely impact pathways; developing and implementing strategies; and designing and implementing monitoring programs. Recent research presents the SIA Framework for Action as a tool for improving social development outcomes (e.g., community resilience) in post-disaster and vulnerable regions through co-production of transformative knowledge [33]. The World Bank has created a guide to adapt SIA to a post-disaster context, more narrowly focusing in on socio-economic impacts, impacts on social relationships and cohesion, impacts on community risks and institutions, and the impacts of the patterns of relief and reconstruction on vulnerability, equity, and decision making and resolution of problems related to the implementation of relief and recovery efforts [117]. More research is needed to determine whether this tool is able to accurately predict and mitigate post-disaster issues, like those raised in the case studies.

Intensive technical assistance is a third tool needed to increase the awareness and use of appropriate types of assessment techniques (e.g., those including metrics of impacts on equity). If alternative metrics had been considered in Puerto Rico before making the decision to close a third of the schools, for example, an alternative solution may have arisen that would have resulted in increased equity especially for rural students. Similarly, if Sri Lanka has used alternative metrics in their assessment when considering the buffer zone policy, issues of gentrification due to hotel rebuilding could have been identified earlier. In New Zealand, assessing the impact of a new governance authority with alternative metrics may have identified how federal support could be fine-tuned according to availability of organized local support. Formalizing and supporting a process for equity impact assessment as part of federal grants for rebuilding would potentially prevent unintended outcomes. For example, grant recipients could be formally required to show how they are implementing strategies that reduce negative socio-economic impacts, health outcomes, or other disparities.

A fourth and final example of formalizing mechanisms for assessing and addressing inequities is to create tools to shift capital towards investments that address sustainable development to help nations, cities, and municipalities raise much-needed finance at scale for recovery investments [118]. For example, ethical debt instruments have been growing in popularity over the past five years. Ethical debt instruments channel investor funds into projects that are ethically or socially responsible. In 2017, the World Bank released social development goal bonds to be channeled into projects aimed at eliminating extreme poverty [119]. Green bonds are tied to environmentally friendly investments such as renewable energy and clean transportation [120] and blue bonds fund investments in sustainable ocean industries [121]. GDP-linked bonds and state-contingent debt instruments also allow for a reduction in a debt service during economic downturns, such as post-disaster, that help mitigate costly debt restructuring efforts. Together, these innovations in financing have the potential to promote more sustainable development post-disaster, if appropriately leveraged by affected communities. However, these opportunities are challenged by their relatively small scale, the use of voluntary reporting that can make it difficult to determine the ethical or social value of the investment, and the need for matching funds for some of these opportunities [118].

Many other types of tools could be rallied or innovated to improve how decision makers approach complex societal problems in recovery planning. Determining what tools are most effective for different contexts will need to be evaluated in an iterative, place-based way. An interdisciplinary and collaborative evaluation could be enacted through existing or new research programs. Additional funds, expertise, and other resources will be needed for communities to meet the challenge of more systematic assessments of the impact of these efforts on long-term changes in vulnerability and inequity.

### 4.3. Limitations

The case examples described above are limited to specific post-disaster policies implemented in three island contexts. Although the cases were chosen because they illustrate common challenges faced in coastal communities where resources may be constrained, these cases are not representative of all disaster contexts. A second limitation is that while the selected cases provide concrete examples of observed behaviors, they do not permit us to explain specific underlying mechanisms of policy impacts because of the multiple covarying factors relevant in understanding each situation. Despite these limitations, case examples are one of the best ways to stimulate new research that has immediate implications for real-world decision makers weighing multiple considerations in challenging contexts. Every disaster context has unique characteristics, but identifying lessons learned from the themes repeating across contexts suggests where new or different decision support is needed. Additional research will be needed to evaluate the effectiveness of decision-support tools in specific settings and to establish generalizable findings about how to ensure longer-term vulnerability reduction is a fundamental component of recovery.

## 5. Conclusions

Reactiveness is understandable—and essential—when urgent societal needs must be met immediately following a disaster. However, the nature, pace, and inclusiveness of recovery across a community may be strongly influenced by decisions made early in the recovery process and by local institutional capacity. These early decisions may make longer-term vulnerability and inequity reduction impossible once the wheels of recovery are already in place. Consequently, recovery policies and governance need to be established in a way that allows for a transitional phase and accomplishes long-term strategic goals without unintentionally increasing community vulnerability and inequity. A holistic understanding—based on perspectives from multiple stakeholders—is needed to understand the interacting socio-economic, cultural, environmental, and physical factors that contribute to vulnerability and to ensure that pre-disaster inequities are not amplified in post-disaster futures. A starting point is to ensure mechanisms by which local communities can play a major role in determining both short-term and long-term policies. Exactly how local stakeholders can practically be included in structures and processes at different points in the recovery line needs more deliberation and evaluation. A strict top-down approach to disaster recovery operations is not designed to be inclusive and is vulnerable to elite capture, potentially exacerbating vulnerabilities and inequities at the local community level [34]. Rather, policy and practice need to support local democratic governance, local action and responsibility, and address community needs, capacities, and resilience. Given the non-linear path of recovery efforts, devices are also needed to ensure that policies are revised as new information or changing circumstances emerge. As the above case studies have shown, without comprehensive, iterative approaches to recovery planning and the use of formal mechanisms in some instances, disaster-impacted communities may experience more vulnerability and inequity.
